# Asymmetry Management During 3D-Guided Piezocorticotomy-Assisted MARPE Treatment with Direct Printed Aligners: Case Report

**DOI:** 10.3390/jcm14217773

**Published:** 2025-11-01

**Authors:** Svitlana Koval, Viktoriia Kolesnyk, Daria Chepanova

**Affiliations:** 1 DrKoval Orthodontics, Boca Raton, FL 33431, USA; 2 Department of Obstetrics, Gynecology and Reproductive Sciences, Yale School of Medicine, Yale University, New Haven, CT 06510, USA

**Keywords:** Miniscrew-Assisted Rapid Palatal Expansion, piezocorticotomy, adults, asymmetry, aligners

## Abstract

**Background:** Midpalatal suture expansion is effective in both growing and adult patients, and Miniscrew-Assisted Rapid Palatal Expansion (MARPE) provides greater skeletal effects and fewer dentoalveolar side effects than traditional expanders. However, asymmetric expansion remains a challenge, often influenced by pre-existing craniofacial asymmetries, appliance design, and suture morphology. In this case report, we describe asymmetric expansion with 3D-guided piezocorticotomy-assisted MARPE and its management with directly printed aligners (DPAs). **Methods:** A patient with facial asymmetry, a narrow maxillary arch, and multiple dentoalveolar deformities underwent pre-treatment evaluation, including root inclination analysis and CBCT imaging. A MARPE appliance with 3D-guided piezocorticotomy assistance was applied; post-expansion orthodontic treatment was digitally planned and performed with directly printed aligners. **Results:** During MARPE activation, asymmetric midpalatal suture disarticulation was observed, with greater displacement on the left side due to jackscrew orientation and root proximity. Post-expansion orthodontic correction with DPAs allowed precise root positioning, spatial redistribution, and improved occlusal symmetry. Over 20 months, significant improvements were achieved in midline orientation, axial root inclination, and transverse arch coordination. **Conclusions**: The reported case underscores the importance of pre-treatment evaluation for asymmetries and careful appliance design in MARPE protocols; in addition, it demonstrates that directly printed aligners, supported by digital planning, can provide accurate and efficient dentoalveolar correction following asymmetric expansion.

## 1. Introduction

The literature reports a variety of appliance designs defined as Miniscrew-Assisted Rapid Palatal Expanders (MARPEs). Most cohort studies describing the effects of MARPE refer to Maxillary Skeletal Expanders (MSEs) [1,2,3]. According to the existing literature, the use of an MSE is associated with significant changes in bone structure surrounding the maxillary complex, including an increase in zygomaticomaxillary width, with the center of rotation of the zygomaticomaxillary complex at the proximal point of the zygomatic process of the temporal bone [2].

Maxillary suture disarticulation patterns were described in a review article by Zarate-Guerra et al. [4], who concluded that, with Miniscrew-Assisted Rapid Palatal Expansion appliances, the symmetry of perimaxillary suture disarticulation depends on pterygomaxillary suture separation and its degree.

The symmetry of expansions with MARPE and RPE was compared by Barton and coauthors [5], in a study of 180 CBCT scans of 60 growing patients. The authors concluded that, besides greater degrees of dentoalveolar effects on molar inclination, both techniques were associated with no significant asymmetries.

Furthermore, a series of case reports shows the efficiency of MARPE in treating pre-existing unilateral and posterior cross-bites, the most widespread asymmetrical conditions diagnosed during the pre-treatment stage [6,7,8].

ALmaqrami and colleagues published a clinical trial evaluating factors associated with asymmetrical expansion through MARPE treatment in adult patients [9]; their study linked asymmetrical expansion with pre-existing asymmetry of the midpalatal suture. The authors reported a 46% incidence of asymmetrical expansion.

Another research group studied fronto- and nasomaxillary suture disarticulation effects in adult patients treated with tooth–bone-borne expanders [10]. The incidence of asymmetry in this study was 30% and was primarily associated with the unilateral asymmetric separation of the frontomaxillary suture and pre-existing facial asymmetry.

The recently introduced 3D-guided midpalatal piezocorticotomy-assisted MARPE treatment protocol presents improvements regarding the predictability of Midfacial Expansion in adult patients and the symmetry of nasal floor separation [11], it allows to eliminate the factor of midpalatal suture shape and direction variability on the outcomes of MARPE. The recently introduced guided midpalatal piezocorticotomy technique additionally eliminates the impact of the midpalatal suture maturation stage and preserves nasal septum position, while following its attachment to the maxillary crests.

The objective of the current case report is to analyze the source of the asymmetry that occurred during a 3D-guided midpalatal piezocorticotomy-assisted MARPE treatment and describe the treatment sequence attributed to its correction. The purpose of this case report is to evaluate the factors contributing to post-expansion maxillary asymmetry.

## 2. Materials and Methods

### 2.1. Pre-Treatment Records and Analysis

A 32-year-old female patient, with non-significant past medical history, relatively healthy, social history negative for smoking, with no reported medications, allergies, past surgical interventions presented for consultation with the chief concern of needing to restore adequate tooth spacing and inter-arch relationships due to the presence of multiple microdontia that would be the focus of future restorative work. Pre-treatment analysis was conducted to ensure that comprehensive treatment planning, restoration of functional occlusion with canine guidance, esthetic positioning of anterior teeth, and enough clearance for potential restorative work would be provided.

### 2.2. Pre-Treatment Diagnosis

**Figure 1:**
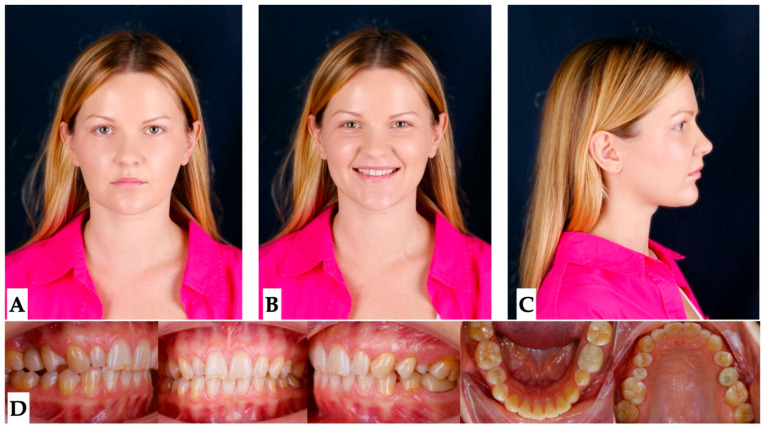
Pre-treatment extra- and intraoral photographs showing facial asymmetry, with chin shifted to the left side, mandibular midline shifted 2.5 mm to the left compared to the maxillary dental midline, which coincides with the facial midline. Occlusal intraoral photographs show multiple microdontic teeth, including peg-shaped #7, #10, microdontic #13, #20, missing #21, and implant crown #4. (**A**)—extra-oral view in repose, (**B**)—extra-oral view smiling, (**C**)—extra-oral profile, (**D**)—intra-oral views.

**Figure 2:**
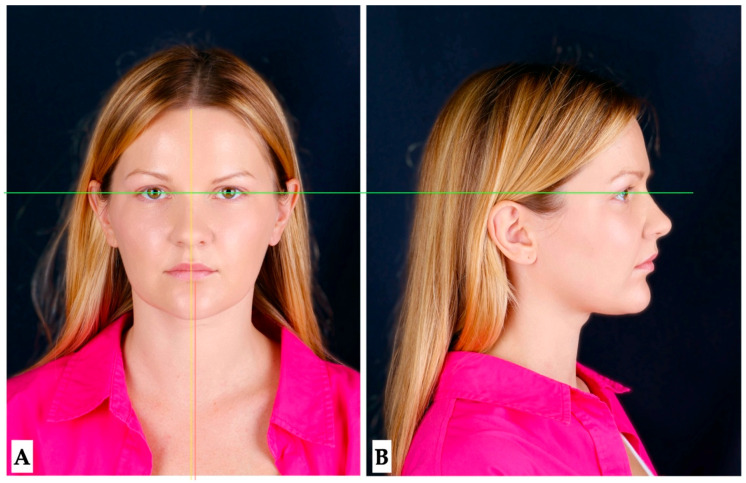
Pre-treatment extra-oral photographs showing facial asymmetry with chin shifted to left side (**A**), and retruded profile (**B**). The face was oriented relative to the true horizontal line (green), Midfacial midline and the midline (yellow) of the lower third of the face (pink) did not coincide.

**Figure 3:**
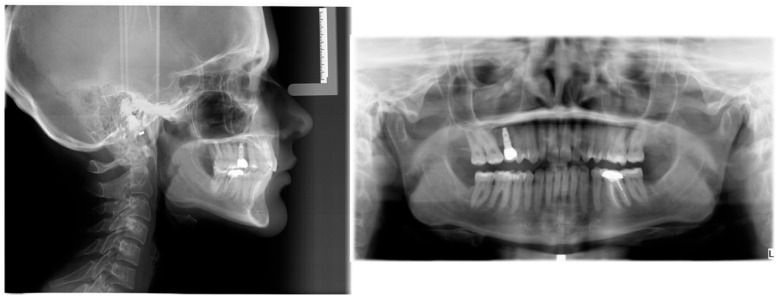
Pre-treatment lateral cephalometric radiograph taken in habitual occlusion, panoramic radiograph taken with a standard bite block technique.

**Figure 4:**
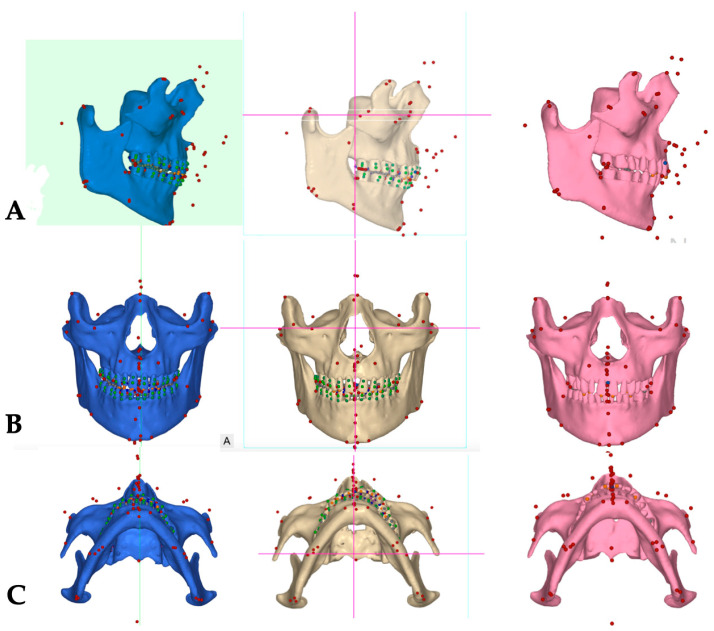
Pre-treatment (blue), post-expansion (beige), and post-aligner (pink) CBCT volume orientation in three spatial planes. (**A**)—Orientation in the sagittal plane with the Orbito-Condylion plane as a reference; (**B**)—coronal plane orientation with the Orbital plane as a reference; and (**C**)—axial plane orientation with the ANS-PNA plane as a reference.

Pre-treatment extra- and intraoral photographs, along with the CBCT, were analyzed to determine the presence of any pre-existing skeletal, dentoalveolar, and dental asymmetries (Figure 1, Figure 2, Figure 3 and Figure 4).

**Facial analysis**: Pre-existing facial asymmetry, with the lower third of the face midline (chin alignment) shifted to the left side, was noted. Reduced lower third of the face height and retruded profile were distinct features of the pre-treatment facial analysis (Figure 1 and Figure 2).

**Skeletal analysis**: The Orbito-Condylion line was used to orient the maxillomandibular complex before and after expansion [12] in the sagittal plane; the Orbital plane was used for coronal plane orientation; and the ANS-PNS plane was used to orient the pre-treatment maxillomandibular complex in the axial plane. The limitation of the pre-treatment CBCT record is that the patient was not maintaining habitual occlusion. Pre-treatment, post-expansion, and post-aligner CBCT volumes were oriented relative to the Orbital-Condylion and Orbital planes for the consistency of superimpositions, measurements, and comparisons (Figure 4).

For the consistency of measurements, all further DICOM volumes (post-expansion and post-aligner) were rendered as maxilla, mandible, and tooth meshes, relative to the Orbito-Condylion plane in the sagittal orientation and to the Orbital plane in the coronal orientation. The DICOM volume orientation, 3D cephalometric measurements, and superimpositions were made in NemoFAB software 2025 (Nemotec, Madrid, Spain). Modified Arnett 3D cephalometric analysis was applied to all 3D cephalometric records [13,14].

The 3D cephalometric measurements are described in Table 1. Pre-treatment, post-expansion, and post-aligner measurements are compared in Table 2.

**Figure 5:**
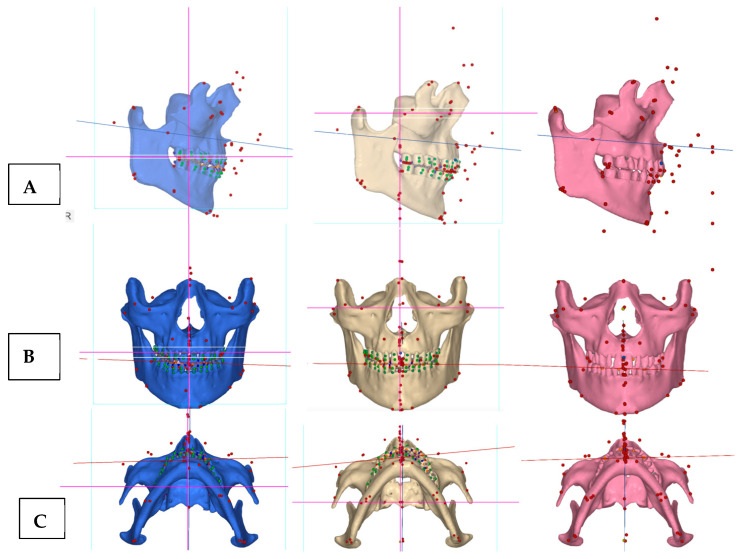
Pre-treatment (blue), post-expansion (beige), and post-aligner (pink) CBCT volume orientation in three spatial planes. (**A**)—Orientation in the sagittal plane with the Orbito-Condylion plane as a reference; (**B**)—coronal plane orientation with the Orbital plane as a reference; and (**C**)—axial plane orientation with the ANS-PNS plane as a reference line. ANS-PNS planes and maxillary canine lines are shown to depict the impact of maxillary expansion on the antero-posterior orientation of the left dentoalveolar process anteriorly to the transverse suture, causing anterior rotation of the maxillary canine line on the left side in the axial plane (beige, post-expansion stage).

Figure 5 shows the orientation of the ANS-PNS plane in sagittal orientation during all three phases of treatment, while the maxillary canine plane is shown to reflect the impacts of midpalatal and unilateral transverse suture disarticulation on the antero-posterior maxillary arch symmetry and concentration of the effects of unilateral transverse suture disarticulation in the axial projection.

**Figure 6:**
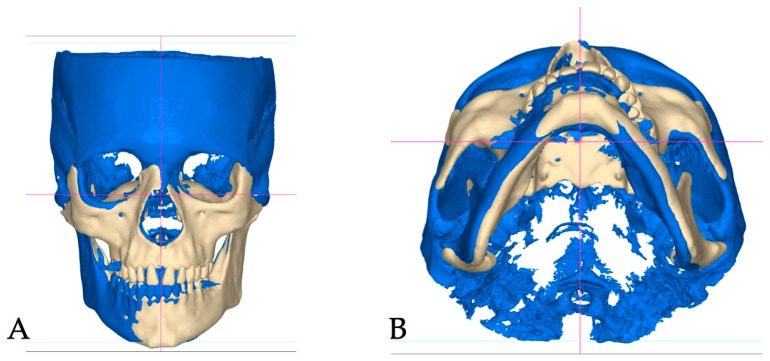
Pre-treatment (blue) and post-expansion (beige) renderings superimposed and shown in coronal (**A**) and axial–caudal (**B**) projections. The axial–caudal view clearly shows asymmetrical displacement of the left maxillary dentoalveolar process, along with the left palatal process of the maxillary bone, due to asymmetrical disarticulation of the transverse suture.

**Figure 7:**
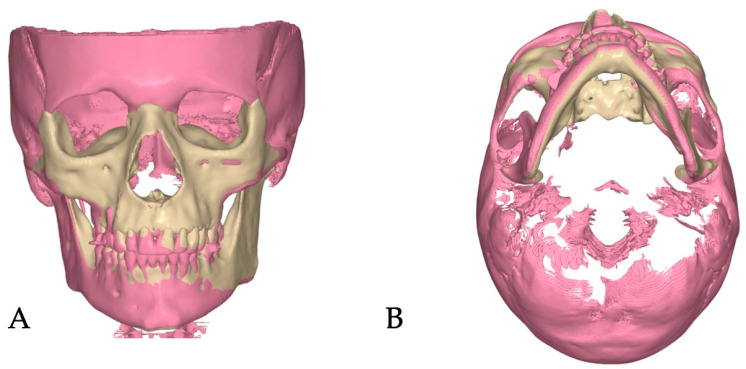
Post-expansion rendering (beige) superimposed over post-aligner (pink) rendering and shown in two projections ((**A**)—coronal, (**B**)—axial–caudal) showing greater impact of the directly printed aligner treatment on compensation of the left palatal process drift due to asymmetric disarticulation of the transverse suture.

Superimpositions of the pre-treatment (blue mesh) and post-expansion (beige) (Figure 6) renderings, along with the post-expansion (beige) and post-aligner (pink) (Figure 7) renderings, are provided to showcase the effects of expansion and treatment.

**Figure 8:**
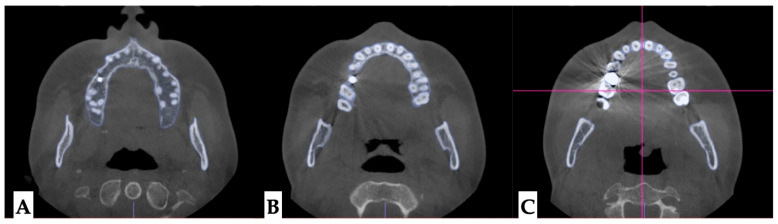
Pre-treatment axial plane visualizations. (**A**)—Axial slice at the level of the ANS, with the skull oriented to all three reference planes; this view shows closer proximity of the left upper quadrant roots to the buccal cortical plate of the alveolar process. (**B**)—Axial view in the same orientation at the level of the 1/2 root length, following the same pattern of buccal cortical plate proximity of the UL maxillary roots. (**C**)—ANS-PNS and Orbital plane orientation at the level of the lower 1/3 of the root length. The vertical line represents midfacial plane in axial projection with the horizontal line being the line perpendicular to the midfacial vertical constructed through the middle of the maxillary right first molar.

**Figure 9:**
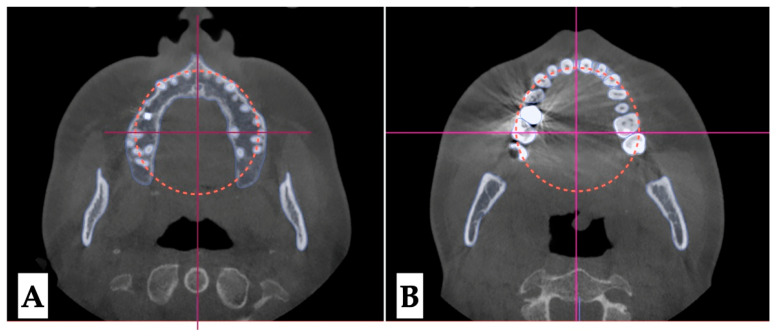
Pre-treatment axial plane visualizations. (**A**)—Axial slice at the level of the ANS, with the skull oriented to all three reference planes; this view shows closer proximity of the left upper quadrant roots to the buccal cortical plate of the alveolar process. (**B**)—ANS-PNS and Orbital plane orientation at the level of the lower 1/3 of the root length. Both views show asymmetry in UL root positioning relative to the constructed circle, with vertical diameter coinciding with the ANS-PNS plane. The vertical line represents midfacial plane in axial projection with the horizontal line being the line perpendicular to the midfacial vertical constructed through the middle of the maxillary right first molar.

**Dentoalveolar analysis:** Dentoalveolar slicing was performed at different levels of the maxillary tooth roots (apical third, half of the root length, and lower third of the root length) to evaluate root position symmetry relative to the buccal cortical plate of the maxillary alveolar process. The upper left quadrant showed clear asymmetry of the root positions, with closer proximity to the buccal cortical plates. (Figure 8 and Figure 9). Palatal plane orientation before treatment was parallel to the constructed Orbital plane, as seen in Figure 8, while the maxillary occlusal plane shows definitive canting, with the left-side occlusal plane located below that of the right side.

**Figure 10:**
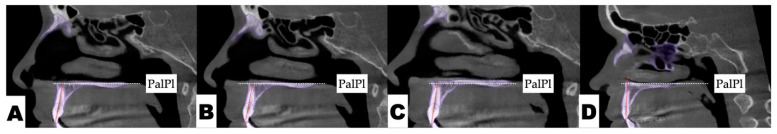
The sagittal view of the maxillary incisor axial cross-section. (**A**)—Maxillary right lateral incisor; (**B**)—maxillary right central incisor; (**C**)—maxillary left central incisor; (**D**)—maxillary left lateral incisor. The axial inclinations of the maxillary left incisors are steeper compared to those on the right side. PalPl- Palatal Plane, the line constructed through ANS and PNS.

**Dental analysis**: Differential inclination of the maxillary incisors is shown in Figure 10.

### 2.3. Procedures and Appliances

#### 2.3.1. Surgical Protocol

All procedures were performed under local anesthesia, initiated with topical application of 20% benzocaine, followed by infiltration of 0.5% bupivacaine (Marcaine) with 1:200,000 epinephrine into the mucosa overlying the midpalatal suture and surrounding tissues.

#### 2.3.2. Design of 3D Surgical Guide

A patient-specific surgical guide was fabricated using NemoCast software 2025 (Nemotec, Madrid, Spain). The guide was designed according to the anatomical position of the nasal septum in the sagittal, coronal, and axial planes.

#### 2.3.3. Osteotomy Planning and Appliance Design

**Figure 11:**
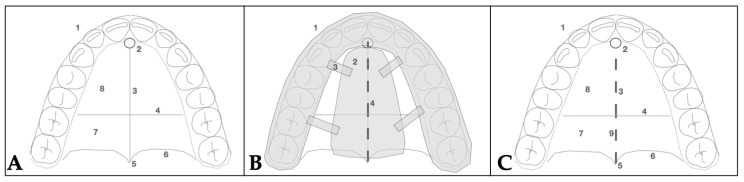
3D guide planning stages for the midpalatal piezocorticotomy: (**A**)—orientation of the main structures of the hard palate (1—dental arch, 2—incisive foramen, 3—midpalatal suture, 4—transverse suture, 5—point PNS, 6—posterior border of the palatine bone, 7—transverse process of the palatine bone, 8—palatal process of the maxillary bone), (**B**)—schematic image of the constructed guide, (**C**)—location of the incision (9—piezocortical incision location following application of the 3D guide).

Piezocorticotomy cuts were individually planned for the patient to preserve approximately 10–12 mm of intact midpalatal suture anteriorly near the incisive foramen to prevent neuro-vascular damage. The cuts extended posteriorly toward the posterior nasal spine (PNS), enabling midsagittal separation of the maxillary palatal processes and the horizontal plates of the palatine bones. The design of the midpalatal 3D guide was described in an earlier publication [11] and accounts for root proximity, incisive foramen location, and, mainly, the orientation of the nasal septum attachment to the maxillary crests in the axial orientation, which does not necessarily follow the orientation of the mucosal outline of the midpalatal suture, leaving purely visual landmarks for performing midpalatal auxiliary disarticulation techniques. The choice of the 3D-guided midpalatal piezocorticotomy-assisted technique combined with MARPE was made to avoid the limitations of the midpalatal suture orientation, preserve nasal septum position, and ensure symmetrical and even disarticulation, as well as to allow for minimal side effects related to peri-maxillary suture-contributing resistance decreasing the potential of the MARPE appliance capacity (Figure 11).

**Figure 12:**
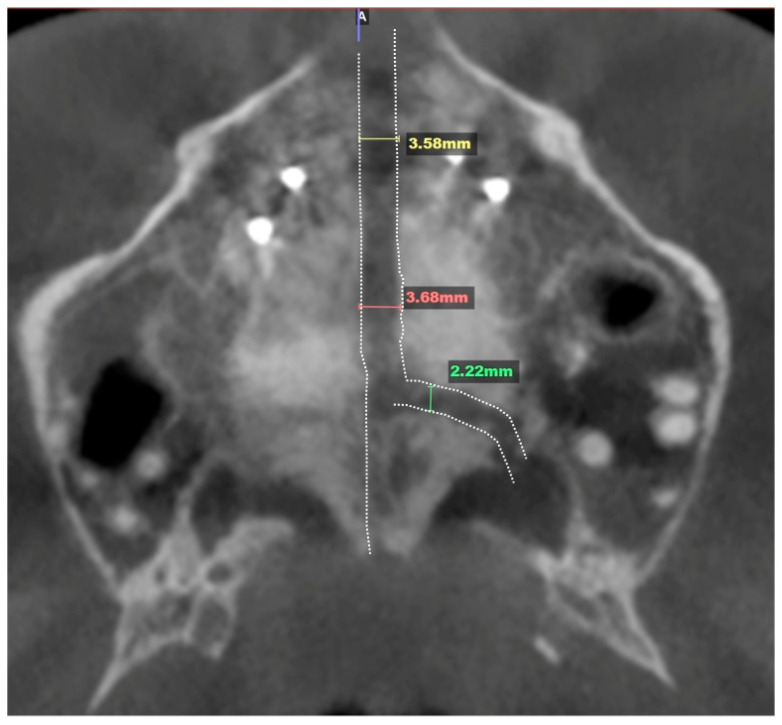
Midpalatal separation in the amount of 3.6 mm with the unilateral transverse suture disarticulation in the amount of 2.22 mm on the left side. The dotted line represents the outline of suture separation.

The custom 3D-printed MARPE appliance was fabricated incorporating a 12 mm Powerscrew (Tigerdental, Horbranz, Austria) and 6 anchoring miniscrews (4 × 1.8 × 15 mm, 2 × 1.8 × 13 mm, Biomaterials Korea, Seoul, Republic of Korea). The Tiger screw activation protocol was 1 turn/day, equaling to 0.11 mm/day, for the total of 8 weeks with the total number of reported 52 turns. The total amount of midpalatal suture disarticulation was 3.6 mm and left side transverse suture separation in the amount of 2.4 mm. It is shown in Figure 12.

#### 2.3.4. Postoperative Assessment, Outcome Analysis, and Asymmetry Correction

**Figure 13:**
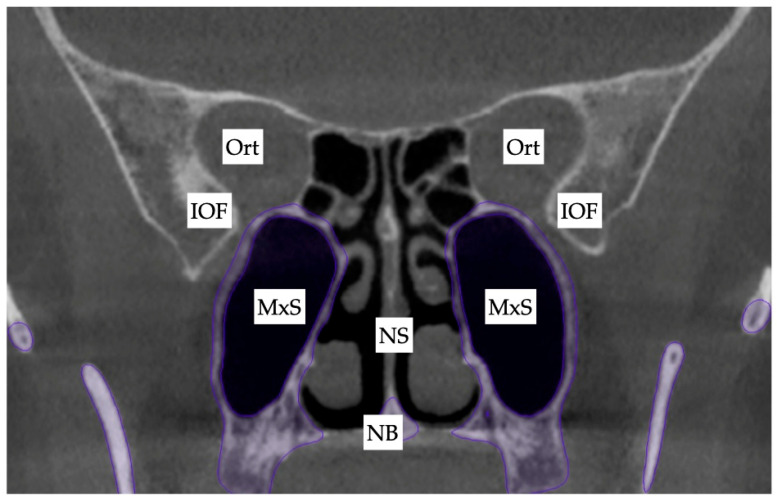
The initial inclination (yaw) of the maxillary base at the level of the maxillary sinus posterior wall and the inferior orbital fissure. Ort—Orbit; IOF—inferior orbital fissure; MxS—maxillary sinus; NS—nasal septum; NB—nasal base.

Due to the tooth–bone-borne nature of the appliance, its placement is limited by the shape of the hard palate and its outline, the yaw of the palatal (maxillary base) plane, and its relationship to the plane connecting the posterior–superior edges of the pterygomaxillary fissure (Figure 13).

**Figure 14:**
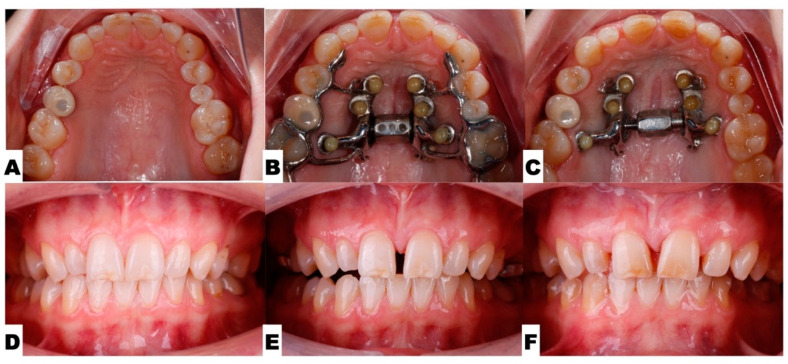
Process of 3D-guided midpalatal piezocorticotomy-assisted MARPE. (**A**,**D**)—Pre-expansion occlusal and frontal views. (**B**,**E**)—Expansion at 4 weeks after 3D-guided midpalatal piezocorticotomy-assisted MARPE; activation 1 turn/day (0.11 mm/turn). (**C**,**F**)—Post-expansion progress prior to initiation of aligner space closure and alignment.

The following pre-expansion, expansion, and post-expansion maxillary occlusal views show the progress of 3D-guided piezocorticotomy-assisted MARPE. Six miniscrews anchor the MARPE appliance to the bone of the palatal processes of the maxillary bones, with the framework cemented to the maxillary molars, premolars, and canines, including implant crown #4 (Figure 14).

**Figure 15:**
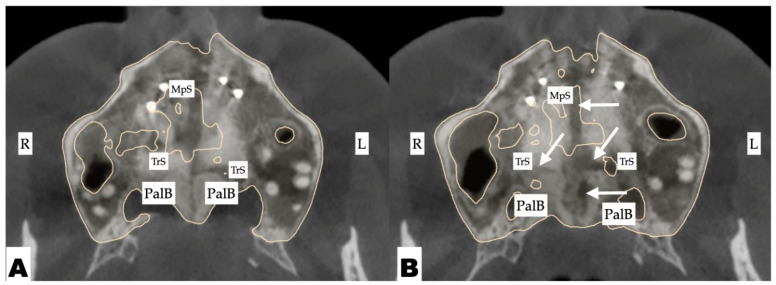
Disarticulation of the midpalatal and transverse sutures with 3D-guided midpalatal piezocorticotomy-assisted MARPE. Both axial slices show disarticulation of the transverse suture, with a greater degree of separation on the left side. Arrows point to areas of disarticulation. R—right side; L—left side; MpS—midpalatal suture; TrS—transverse suture; PalB—palatine bone. (**A**)—caudal axial slice (showing no right-side transverse suture disarticulation and 1.5 mm of left-side disarticulation); (**B**)—rostral axial slice (showing 1 mm of right-side disarticulation and 2.22 mm of left-side transverse suture disarticulation).

The pattern of midpalatal suture separation was evaluated after the completion of expansion and is shown in Figure 15. The midpalatal suture was completely disarticulated, involving both the ANS and PNS areas. The transverse palatal suture showed signs of comparable disarticulation on the left side resulting in complete separation of the palatine process of the maxillary bone on the left side and its migration forward and outward due to appliance activation.

**Figure 16:**
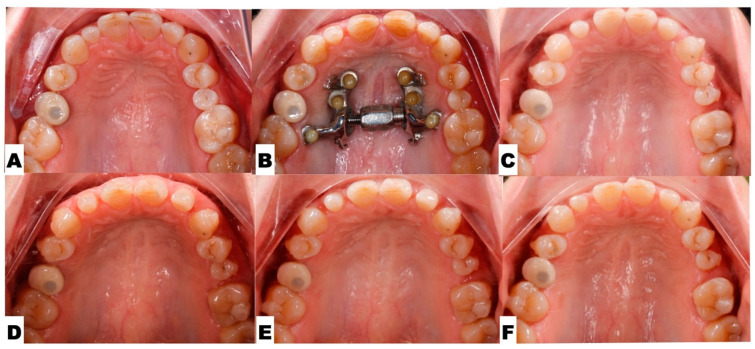
The progression of post-expansion aligner dentoalveolar movements, with redistribution of teeth along the dentoalveolar process for further restorative treatment of microdontia. (**A**)—Pre-expansion; (**B**)—immediately after completion of 3D-guided midpalatal piezocorticotomy-assisted MARPE; (**C**)—distalization of the left posterior teeth initiated with space opening for restorative treatment of lateral incisors and second maxillary left premolar; (**D**–**F**)—progression of direct aligner-assisted tooth movements for premolar de-rotation, molar distalization, and axial inclination correction for maxillary incisors, with alignment of incisal edges due to complex root torque movements.

**Figure 17:**
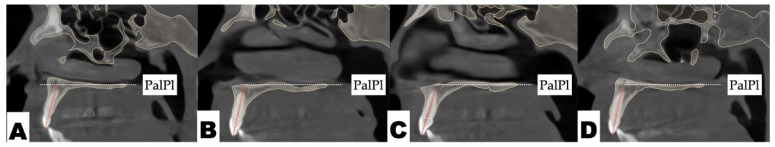
The sagittal view of the axial cross-section of maxillary incisors after aligner axis correction: (**A**)—maxillary right lateral incisor; (**B**)—maxillary right central incisor; (**C**)—maxillary left central incisor; (**D**)—maxillary left lateral incisor. The axial inclinations of all maxillary incisors have improved, with significant reductions in buccal root torque in the maxillary left central and lateral incisors with directly printed aligner orthodontic movement (Table 3).

Post-expansion dentoalveolar movement progression is shown in Figure 16. The initial axial inclinations of maxillary incisors, microdontia with varying cross-sections of the maxillary incisor roots, and the pattern of disarticulation of the transverse suture all contributed to the degree of post-expansion incisor position asymmetry, with perceived dominance of the buccal outline of the left maxillary segment compared to the right side. The dentoalveolar tooth positions, along with the differential initial root inclinations, were progressively corrected with directly printed aligner orthodontic appliances (Thera-Hartz TA-28, Graphy, Seoul, Republic of Korea). Post-alignment axial inclinations of the maxillary incisors are shown in Figure 16 and Figure 17.

**Table 3:** Contains maxillary incisor inclination changes with DPA treatment.

Tooth	Inclination Before Expansion, Degrees	Inclination After Expansion, Degrees	Difference, Degrees
Max Left Lateral Inc	92	101	+9
Max Left Centr Inc	99	108	+9
Max Right Centr Inc	101	102	+1
Max Right Lat Inc	100	106	+6

**Figure 18:**
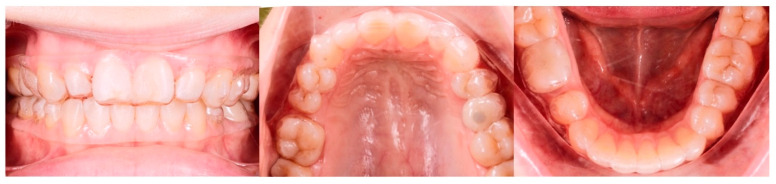
Directly printed aligners (Thera-Harz, TA-28 resin, Graphy, Seoul, Republic of Korea) in post-expansion stage.

Figure 18 shows the directly printed aligners during the pre-restorative stages of treatment.

### 2.4. Pre-Restorative Records

**Figure 19:**
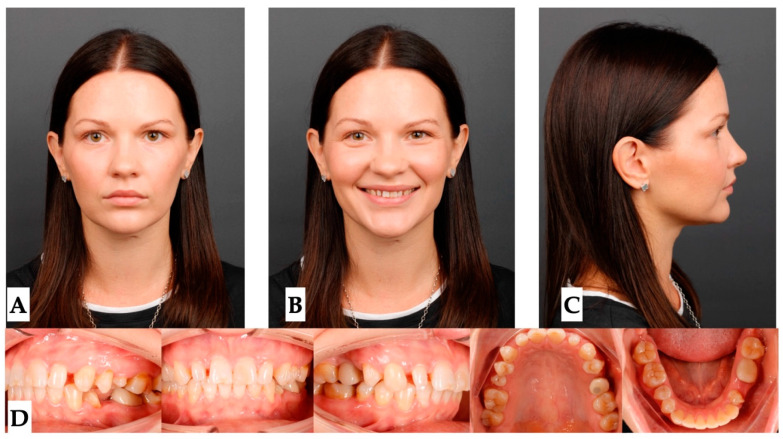
Post-treatment extra- and intraoral photographs showing facial symmetry, with coinciding facial, maxillary, and mandibular midlines. Occlusal intraoral photographs show multiple microdontic teeth, including peg-shaped #7, #10, microdontic #13, #20, missing #21, and space re-opened for implant crowns #21 and #4, and extra space for restoration of microdontic teeth: (**A**)—extra-oral view in repose, (**B**)—extra-oral view smiling, (**C**)—extra-oral profile, (**D**)—intra-oral views.

The pre-restorative records are shown in Figure 19.

## 3. Results

Table 2 contains pre-treatment, post-expansion, and post-aligner 3D cephalometric measurements.

Several measurements represent antero-posterior distances from the landmarks located at the ANS, maxillary incisors, maxillary canines, and maxillary molars. Negative values indicate the caudal location of the landmarks relative to the True Vertical Plane. All the values remained in negative ranges throughout this study, with several values decreasing with treatment. The majority of dental landmarks reduced their distances to the TVL due to mesial movement with directly printed aligners, including the maxillary right and left canines, as well as the maxillary right and left first molars. Post-expansion values indicated reductions in the distances from the TVL to the maxillary left canine and left first molar due to the asymmetric nature of maxillary skeletal expansion and contributing left transverse suture separation.

The cant of the maxillary canine line reduced with expansion and remained at the same level after DPA treatment.

Expansion favored the left side in the context of maxillary first molar width difference, as well as in zygomatic arch and lateral orbital rim widths, the latter of which reduced with DPA treatment.

Vertical distances from the maxillary incisor and maxillary canines, measured from the True Horizontal Line (THL) oriented through the Nasion, were reduced with DPA treatment, indicating a relative intrusion due to root inclination changes and active remodeling in the area of the ANS. Incisor axis inclination relative to the ANS-PNS plane were increased for all four maxillary incisors with the highest impact in the area of maxillary left central and lateral incisors (+9 degrees of crown buccal inclination).

The orientations of both the ANS-PNS and maxillary canine lines showed changes with expansion. Thus, the ANS-PNS plane rotated counter-clockwise, as seen in Figure 5, while the maxillary canine line rotated anteriorly out on the left side in the axial projection after expansion and was further corrected with DPA treatment.

## 4. Discussion

Multiple studies have shown the efficacy of midpalatal suture separation with both Rapid Palatal Expanders (RPEs) and MARPEs in growing individuals, emphasizing greater stability during the consolidation stage and lesser bending effects on alveolar processes with MARPEs [15,16]. Other studies have shown successful MARPE applications in adult patients, emphasizing careful planning for the success of midpalatal suture disarticulation [17], as well as appliance design and activation planning regarding the maxillary center of resistance that can control maxillary rotation during expansion [18].

The current case study describes an occurrence of asymmetric expansion with 3D-guided midpalatal piezocorticotomy-assisted MARPE and its management with post-expansion orthodontic movements using DPAs. The post-expansion phase in the current case study is marked by unilateral anterior displacement of the left palatal process of the maxillary bone, associated with unilateral disarticulation of the transverse suture. The post-expansion orientation of the jackscrew favored an anterior displacement vector on the left side of the palate. Screw placement, asymmetric initial position of the maxillary first molars, the presence of multiple microdontic teeth, and a dental implant could be contributing factors. In addition, pre-treatment root inclination of the maxillary central and lateral incisors, and canine, also impacted the perception of maxillary antero-posterior asymmetry in the post-expansion phase.

Post-expansion orthodontic treatment lacks descriptive studies and is limited to case reports. Bud and colleagues described side effects of MARPE with and without corticopuncture [19] and reported occlusal modifications occurring post-expansion that are related to asymmetrical occlusal relationships prior to the beginning of treatment.

The present study focuses on the incidence of the post-expansion asymmetry, presumably caused by the appliance design and further aggravated by the close proximity of the roots of the maxillary incisors on the left side. Initial root orientations were evaluated as a part of the staged analysis, revealing differential root inclination of the maxillary incisors relative to the buccal cortical plate of the maxillary alveolar process. Root proximity and higher buccal root torque of all maxillary incisors, with the left incisors being in closer proximity compared to the right side, was one of the predisposing factors to pre-existing asymmetry of the maxillary anterior alveolar process in the axial plane.

As Almagrami and coauthors have previously pointed out [9], midpalatal suture asymmetry is one of the factors most frequently associated with asymmetrical MARPE. As stated in the case report by Choi and coauthors [18], appliance design can influence the force vectors applied during maxillary skeletal expansion with a MARPE appliance. The above two studies are in agreement with the present case report, indicating the impacts of both pre-existing asymmetries and appliance design in asymmetric expansion outcomes.

Post-expansion dentoalveolar correction was performed using directly printed aligners (DPAs). The efficacy of DPAs has been demonstrated in multiple studies [20,21,22], which describe their higher accuracy, precision, efficacy, and shape-memory effects, along with higher degrees of root control, due to the thermoelasticity of the Thera-Hartz resin and appliance design that incorporates longer margins. The choice of DPAs was made due to the eliminated need for attachments and the higher degree of root control necessary for the indicated palatal root torque correction. The extended margin of the aligners delivers forces closer to both center of rotation and center of resistance of the tooth. NemoCast (Nemotec, Madrid, Spain) planning software was used to plan orthodontic tooth movements and enable precise positioning of the roots by merging intraoral and CBCT scans. Aligners were 3D printed using Graphy Thera-Hartz TA-28 resin and compatible 3D printers in the office.

Improvements in midline orientation, axial root inclination, and spatial redistribution were achieved over the course of 20 months post-expansion. Axial root inclinations of all four maxillary incisors were improved from 1 to 9 degrees of buccal crown torque. Reduction in both canine line rotations in the axial plane, with preservation of their vertical orientations, was achieved. Mesial movements of the maxillary teeth were predictably executed to redistribute space for further restorative intervention.

Study limitations included the lack of the habitual occlusion in the pre-treatment CBCT records with subsequent inability to evaluate mandibular condyle positional changes with treatment. Inadequate bone amount in the distal third of the palate accounted for the interradicular placement of the MARPE screws.

The current case report presents insights into factors contributing to the development of post-expansion asymmetry with custom-made MARPE appliances. While 3D-guided midpalatal piezocorticotomy is claimed to preserve nasal septum orientation, as well as allow for even and symmetrical disarticulation of the midpalatal suture, the appliance design and the presence of predisposing factors may contribute to the development of asymmetry in other spatial planes.

Directly printed aligners were shown to be effective means of correcting dentoalveolar discrepancies and mitigating the effects of post-expansion asymmetry due to asymmetrical transverse suture separation in the sagittal plane.

## 5. Conclusions

The reported case study highlights the efficacy and challenges of MARPE treatment, particularly when asymmetry occurs due to factors such as midpalatal suture orientation, root proximity, and appliance design. The patient presented with pre-existing facial and dental asymmetries that contributed to uneven transverse expansion, with greater displacement on the left side. Post-expansion correction using directly printed aligners (DPAs) allowed precise root control and effective spatial redistribution, supported by digital planning tools. Over 20 months, treatment achieved significant improvements in facial symmetry, root inclination, and was able to eliminate the effects of post-expansion asymmetry.

## 6. Patents

US and Canada Patent Pending: Piezocorticotomy guide for midpalatal skeletal expansion (Application # 18/919,416).

## Figures and Tables

**Table 1:** Descriptive characteristics of the 3D cephalometric measurements used in this study.

Measurement	Description
ANS projection to True Vertical Line (TVL)	Horizontal distance from ANS to TVL (negative values indicate location caudal to TVL, while positive values indicate rostral to TVL)
Mx Incisor R to TVL	Horizontal distance from the right maxillary central incisor to TVL
Mx Incisor L to TVL	Horizontal distance from the left maxillary central incisor to TVL
Upper Right Canine to TVL	Horizontal distance from the right maxillary canine to TVL
Upper Left Canine to TVL	Horizontal distance from the left maxillary canine to TVL
Upper Right Molar to TVL	Horizontal distance from the right maxillary first molar to TVL
Upper Left Molar to TVL	Horizontal distance from the left maxillary first molar to TVL
Mx33 Cant	Maxillary canine line cant
Mx Molar width Difference	Difference from the right maxillary first molar to the maxillary midline compared to the distance from the left maxillary molar to the maxillary midline (positive values indicate the right-side value is higher than the left-side value)
Zyg Arch	Difference in right zygomatic arch distance to the maxillary midline compared to the left zygomatic–maxillary midline distance
Lat Orb Rim	Difference in right lateral orbital rim distance to the facial midline compared to the left lateral orbital rim distance
Mx Incisor to THL (Na)	Projection from the incisal edge of the maxillary central incisor to the True Horizontal Line through Na
Mx Canine R	Projection from the incisal edge of the maxillary right canine to the True Horizontal Line through Nasion
Mx Canine L	Projection from the incisal edge of the maxillary left canine to the True Horizontal Line through Nasion
Mx Molar R	Distance from the Right Maxillary Molar Yaw Point to the Midfacial Midline
Mx Molar L	Distance from the Left Maxillary Molar Yaw Point to the Midfacial Midline
Zyg Arch R	Distance From the Right Zygomatic Arch to the Midfacial Midline
Zyg Arch L	Distance From the Left Zygomatic Arch to the Midfacial Midline
Lat Orb Rim R	Distance from the Lateral Aspect of the Right Orbital Rim to the Midfacial Midline
Lat Orb Rim L	Distance from the Lateral Aspect of the Left Orbital Rim to the Midfacial Midline

**Table 2:** Pre-treatment, post-expansion, and post-aligner measurements derived from the 3D cephalometric tracings of DICOM volumes.

Measurement	Before Treatment	Post-Expansion	Post-Aligner
ANS projection to True Vertical Line (TVL), mm	−9.5	−14.1	−10.2
Mx Incisor R to TVL, mm	−16.1	−16.1	−14.6
Mx Incisor L to TVL, mm	−15.9	−14.7	−14.1
Upper Right Canine to TVL, mm	−22.7	−22.8	−19.9
Upper Left Canine to TVL, mm	−21.9	−19.6	−17.5
Upper Right Molar to TVL, mm	−40.1	−41.6	−38.2
Upper Left Molar to TVL, mm	−37.5	−35.6	−34.0
Mx33 Cant, degrees	−1.0	−0.2	−0.3
Mx Molar width Difference, mm	4.0	−2.4	−3.1
Zyg Arch, mm	1.7	−1.3	−2.8
Lat Orb Rim, mm	1.2	−1.3	−0.9
Mx Incisor to THL (Na), mm	69.7	69.8	61.2
Mx Canine R, mm	68.5	69.2	60.6
Mx Canine L, mm	69.5	69.5	60.9
Mx Molar R, mm	30.1	28.1	28.0
Mx Molar L, mm	26.1	30.5	31.1
Zyg Arch R, mm	54.5	54.2	53.3
Zyg Arch L, mm	52.8	55.6	56.1
Lat Orb Rim R, mm	51.2	50.5	50.9
Lat Orb Rim L, mm	50.0	51.8	51.8

## Data Availability

The original contributions presented in this study are included in the article. Further inquiries can be directed to the corresponding author.

## Abbreviations

The following abbreviations are used in this manuscript:

MARPE	Miniscrew-Assisted Rapid Palatal Expansion
ANS	Anterior Nasal Spine
PNS	Posterior Nasal Spine
DPA	Directly Printed Aligner
CBCT	Cone-Beam Computer Tomography
MSE	Maxillary Skeletal Expander
